# “Make sure you involve us” – Youth experiences, care needs, and areas for intervention across the TB care journey in East Africa: a qualitative study

**DOI:** 10.21203/rs.3.rs-10969224/v1

**Published:** 2026-10-07

**Authors:** Leslie A. Enane, Lameck Diero, Winnie Muyindike, MaKayla Waugh, Kirsten Prabhudas-Strycker, Sylvia Kitur, Julia Omondi, Elyne Rotich, Sarah Namwanje, Lillian Ayesiga, Alexis Byaruhanga, Bob Ssekyanzi, Harold Kooreman, Suzanne Goodrich, Helen Byakwaga, Aggrey Semeere, Neelima Navuluri, Kara Wools-Kaloustian

**Affiliations:** Indiana University School of Medicine; Moi University College of Health Sciences; Mbarara University of Science and Technology; Indiana University Indianapolis; Indiana University School of Medicine; Academic Model Providing Access to Healthcare (AMPATH); Academic Model Providing Access to Healthcare (AMPATH); Academic Model Providing Access to Healthcare (AMPATH); Mbarara Regional Referral Hospital; Mbarara Regional Referral Hospital; Mbarara Regional Referral Hospital; Mbarara Regional Referral Hospital; Indiana University Richard M Fairbanks School of Public Health; Indiana University School of Medicine; Mbarara University of Science and Technology; Makerere University; Duke University School of Medicine; Indiana University School of Medicine

**Keywords:** adolescents, tuberculosis, post-tuberculosis, care cascade, qualitative research, health service delivery, youth-friendly services

## Abstract

**Background:**

Tuberculosis (TB) is a major cause of morbidity and mortality for youth (ages 15–24) globally. We comprehensively investigated youth experiences, care needs, and areas for intervention across the TB diagnosis, treatment, and post-TB care cascade.

**Methods:**

Youth with pulmonary TB and healthcare workers providing TB care in Eldoret, Kenya, and Mbarara, Uganda, were purposively sampled. In-depth semi-structured interviews examined factors influencing youth TB care engagement, care needs, and areas for intervention before, during, and after TB treatment. Reflexive thematic analysis was conducted. A youth advisory board supported study implementation.

**Results:**

We conducted 39 interviews, including 25 healthcare workers and 14 youth with TB. Youth median age was 22, 8 (57%) were male, and one was living with HIV. Youth experiences were driven by poor access to care, including for TB testing, treatment support, and post-TB sequelae. School-related factors included risks for TB acquisition, delayed diagnosis, and barriers to adherence, but also opportunities for treatment support. Stigma and limited TB awareness resulted in delayed diagnosis, inadequate treatment adherence, social isolation, and mental health burdens. Experiences of severe illness, post-treatment morbidity, and TB recurrence impacted physical and mental wellbeing. Treatment support and social support were essential, yet were critically undermined by stigma, economic insecurity, and family circumstances. Areas for intervention centered on accessible, person-centered, youth-friendly care, including after TB treatment.

**Conclusions:**

Youth and healthcare workers detailed the landscape of gaps in care, opportunities, and solutions for youth with TB. Dedicated youth-led interventions may improve TB care access, outcomes, and quality of life.

## Background

Tuberculosis (TB) imposes tremendous burdens for youth (ages 15–24 years) globally.^1,2^ An estimated 1.6 million youth developed TB in 2024,^3^ and TB is a leading cause of death among youth in endemic settings.^4^ Even after successful treatment, TB is a major cause of chronic morbidity, resulting in post-TB lung disease (PTLD) and other health sequelae, with potential longer-term impacts for youth wellbeing.^1,5,6^

Youth experience particular challenges in TB care engagement, which impact their health outcomes. Unique life stage and developmental factors have implications for access and adherence to care for TB—a heavily stigmatized condition with a burdensome course of treatment. Barriers to youth care engagement result in delayed TB diagnosis;^7,8^ adherence gaps, and loss to follow-up.^9–14^ These place youth at increased risk for complications including PTLD.^15^ Recent studies have demonstrated lung impairment in the majority of adolescents after completion of TB treatment.^16–19^ Further, youth experience unique vulnerabilities to economic, social, and psychological impacts of TB and its treatment.^6,20,21^ Given the incidence of TB among youth in high-burden countries, post-TB sequelae may represent a major cause of chronic morbidity and reduced quality of life in this age group.^22,23^

Although youth are a distinct population with high morbidity and mortality from TB, unique risk factors, and care engagement challenges, they have been largely neglected in the global TB response.^1,24–26^ The WHO recommends youth-friendly TB services,^27^ however, implementation is hampered by a dearth of research in this age group.^28^ Data regarding youth experiences and care needs are essential for informing dedicated interventions in TB care and emerging post-TB care models. This qualitative study investigated youth experiences, care needs, and areas for intervention across the TB/post-TB care cascade in Kenya and Uganda. These were examined from the perspectives of youth treated for TB and healthcare workers (HCWs) providing TB care to youth.

## Methods

### Study setting

This study was conducted in the East Africa TB Sentinel Research Network (TB-SRN) of the International epidemiology Databases to Evaluate AIDS (IeDEA). TB-SRN is a global prospective study of people aged ≥ 15 with pulmonary TB.^29^ East Africa TB-SRN sites include Moi Teaching and Referral Hospital in Eldoret, Kenya—the flagship facility of the Academic Model Providing Access to Healthcare (AMPATH) Partnership—and Mbarara Regional Referral Hospital in Mbarara, Uganda.^30^ Both are major referral hospitals and academic centers in their respective regions of western Kenya and southwestern Uganda, and host TB clinics under their respective national TB programs. As is typical throughout TB care settings globally, dedicated youth-friendly services had not been implemented at the time of this study.

### In-depth interviews

To understand care engagement experiences and needs, qualitative investigations were undertaken in in-depth interviews, as previously described.^30^ In-depth interviews included youth with TB (ages 15–24 years) and multidisciplinary HCWs providing care to youth with TB. Purposive sampling sought inclusion of youth at each site with variation by age, sex, and HIV status. Interviews were conducted during TB treatment or within 6 months of treatment completion to capture health experiences over this course. Purposive sampling among multidisciplinary HCWs ensured inclusion of a range of roles within TB and HIV services, including clinical officers, nurses, social workers, and peer supporters. All interviews were conducted between February 2024 and March 2025.

Separate, dedicated semi-structured guides were used to interview individuals with TB and HCWs, with sections adapted to participant group and timing (during or after TB treatment). The guides leveraged the socioecological model for exploring barriers and facilitators across the TB care journey, at diagnosis, treatment, and post-treatment stages (Fig. 1).^31^ Dedicated questions queried youth-specific experiences, care needs, and areas for intervention. The guide was iteratively reviewed by the study team and refined prior to implementation. Initial interviews were reviewed in-depth to incorporate minor adjustments to the guide as needed. Interviews were audio-recorded, and translated/transcribed into English, with verification of translations. Transcripts were linked to participant study ID and did not include identifying information. Finalization of interviews was based on the thematic saturation and information power achieved surrounding youth TB care experiences across the care cascade.^32,33^

### Participant questionnaires

Questionnaires with youth assessed their sociodemographic information and living situation, including family/caregiver factors, school attendance, and relationships. TB and HIV history were abstracted from available records and ascertained in questionnaires. A disclosure screening tool was used to confirm youth disclosure status prior to discussion of HIV history.^34^

### Youth advisors

Engagement with the Adolescent and Youth Advisory Board (AYAB) at AMPATH in Eldoret, Kenya, supported study implementation. The AYAB is a standing advisory board of youth with lived experience of HIV and other conditions, who receive training in human subjects research, youth rights, and advocacy.^35^ The AYAB facilitates youth involvement and provides consultation across research stages. Advisors to this study included youth with experiences in TB and/or HIV care. Prior to study initiation, they were consulted regarding youth-friendly approaches to study engagement. During and after the study, they were consulted in discussion around initial findings, dissemination plans, and priorities for TB care and research.

### Analysis

The coding and thematic analysis process included US- and East Africa-based team members, including experts in adolescent health and TB/HIV care and a former youth peer mentor. We conducted a reflexive thematic analysis starting with close reading and discussion of transcripts and considering the researchers’ positionality, assumptions, and influences.^12^ We developed an initial code list of deductive codes, following the interview guide structure, and added inductive codes from the interview narratives. All transcripts were independently coded by at least two researchers using Dedoose, a web-based platform for collaborative mixed-methods research. Initial coded transcripts were closely discussed to reach consensus around code applications and elaboration of the codes. Qualitative themes were identified based on emerging patterns and relationships. Intersecting, overlapping, or diverging findings were used to support interrogation and elaboration of themes, including exploration of varying perspectives by participant group. Themes and sub-themes were refined among the analysis team following an iterative and flexible process. Preliminary themes were discussed with the study teams at each site and with youth advisors, to further verify interpretations. Basic descriptive statistics were used to describe youth characteristics. We followed the Standards for Reporting Qualitative Research in the preparation of this manuscript.^36^

### Ethics

All participants provided informed consent. For minors, parental/guardian consent and youth assent were required. Ethical and regulatory approvals were obtained from the institutional review boards with oversight of the study: Indiana University Institutional Review Board; MTRH/Moi University Institutional Research and Ethics Committee; the Kenya National Commission for Science, Technology and Innovation; Mbarara University of Science and Technology Research Ethics Committee; and the Uganda National Council of Science and Technology.

## Results

We conducted 39 interviews, including 25 HCWs and 14 youth with TB. Among youth, median age at TB treatment initiation was 22, 8 (57%) were male, and one was living with HIV (Table 1). Youth lived primarily in rural (50%) or peri-urban (36%) areas. Six (43%) lived on less than $2.60 US dollars/day. Four (29%) had been attending school; four (29%) had completed school; and six (43%) were not attending school due to lacking funds. Median duration from recalled symptom onset to TB treatment initiation was 3.0 months (interquartile range, IQR 1.6–4.8). All youth successfully completed treatment for the current TB course. Two had experiences with recurrent TB; one was treated for recurrent TB on entry into the cohort, and one was treated for two recurrences after their initial course at study enrollment. Interviews were completed a median 6.6 months from treatment initiation.

Among HCW participants, 16 (64%) were women. Their roles included registered nurses (40%), medical officers (12%), clinical officers (12%), peer supporters/providers (8%), community health promoters (8%), pharmacist (4%), TB coordinator (4%), program leader (4%), and others (8%).

Cross-cutting themes across the TB care journey (from TB diagnosis, treatment, and post-TB health) were identified, along with specific themes at each stage, and areas for intervention.

### Cross-cutting themes across the TB care journey

Youth experiences across the TB care cascade were driven by issues of limited access to care; specific challenges at school; prevalent stigma; burdens of TB illness and its treatment; associated mental health challenges; and the critical roles of treatment support and psychosocial support (Table 2).

Youth confronted significant challenges accessing TB testing, treatment services that met their needs, and management of post-TB sequelae. Economic vulnerability, compounded with costs of illness and treatment, contributed substantially to care access challenges throughout the care cascade. School attendance, particularly in boarding school, posed risks for TB illness, potential delays in diagnosis and barriers to adherence, but also opportunities for treatment support.

Prevalent stigma and limited TB awareness led to prolonged delays in diagnosis, inadequate adherence or treatment interruptions, and ongoing isolation after treatment completion. Mental health challenges were substantial as youth faced both treatment burdens and intense stigmatization. Substance use among some youth increased TB risk and hampered care engagement and recovery. Experiences of severe TB illness, post-treatment morbidity, and TB recurrence impacted physical and mental wellbeing.

Importantly, at this developmental stage when youth are gaining independence yet still reliant on caregivers, treatment support and social support were essential for TB treatment and recovery. Yet these were critically undermined when stigma precluded the disclosure of TB status involved in seeking support. Poverty and family circumstances also impacted the support available to youth.

### Themes by illness stage

#### From illness onset to TB diagnosis

Youth and HCWs described prolonged delays in accessing TB diagnosis, with youth having multiple healthcare contacts before accessing chest X-ray or TB testing. HCW described that youth often had advanced TB illness by the time of TB diagnosis. Underlying reasons for delays included lacking awareness or suspicion about the possibility of TB or being focused on school or work as symptoms persisted. Residing at boarding school away from caregivers posed particular challenges, not only for TB acquisition, but also for delayed care-seeking or diagnosis. Lack of TB diagnostics at initial sites of careseeking prolonged diagnostic delays. Stigma and misunderstandings about TB further contributed. Diagnosis was facilitated when youth were encouraged by caregivers or healthcare workers to seek further evaluation, and when they accessed care at a facility with Xpert testing and/or chest X-ray. Youth and HCW described needs for information and counseling around TB diagnosis, emphasizing that TB is curable.

#### TB treatment

Youth and HCWs described major barriers to adherence and completion of TB treatment, and risks for loss to follow-up and poor outcomes. They described youth risks for stopping treatment early once feeling better. Because of this, having clear health information, counseling, and close follow-up from attentive and compassionate providers was seen as critical, as was ongoing adherence support from a family member, school nurse, or other trusted supporter.

Stigma presented a predominant barrier to TB treatment adherence and completion. Youth commonly faced difficulties taking TB treatment while at boarding school or around peers or even family members. Stigma and treatment burdens resulted in poor mental health and suicidal thinking. Further major barriers included pill burden and side effects, food insecurity, and advanced TB illness. Such challenges were described as particularly acute for youth with TB-HIV coinfection, who commonly presented with TB either at HIV diagnosis or after HIV care disengagement.

Youth were recognized to have unique developmental needs related to health education, follow-up, and adherence support. Participants emphasized that counseling should impart the seriousness of TB and the importance of treatment adherence and completion. They described engagement of a caregiver, school nurse, and/or other supporter as critical to successful treatment completion in youth. Such critical support was undermined by stigma when youth or family members were unable to disclose TB to seek needed support. HCWs strongly encouraged youth and caregivers to disclose TB, for example, to a school nurse or family member best positioned to support adherence.

Youth with significant family-level challenges, including those who were orphaned or street-connected, faced particular care engagement barriers. Lacking treatment support, low health literacy, competing needs, mobility, and poverty significantly impacted completion of treatment.

#### Post-TB health and wellbeing

Experiences after TB treatment completion reflected both recovery of physical health and wellbeing, as well as ongoing challenges. Some youth described relief from physical symptoms and feeling “free” from the stresses and burdens of treatment. They welcomed being able to return to some previous activities. For others, persistent health complaints troubled them and limited their return to their livelihoods. Some struggled to access care or to afford care-seeking or treatment. Similarly, while many described relief from previous mental distress and suicidal thinking, some also described difficulties now overcoming isolation and stigma as well as reconciling what was “lost” during their illness and treatment.

While post-TB care is not yet routine under national programs, participants described post-TB care needs that they wished to see addressed. They endorsed needs for post-TB follow-up, including health evaluation and guidance for possible TB recurrence, PTLD, or other sequelae. They described needs for social support and mental health care as youth sought to resume their social and working lives. They identified needs for financial support, especially as youth tend to be dependent on caregivers, and many come from households in which multiple family members have been affected by TB. This included financial needs for transportation to post-TB health evaluations, as well as access to financial programs that might support their recovery.

#### Areas for intervention

Multiple opportunities were identified for enhancing youth TB care access, supporting TB treatment, and addressing post-TB care needs (Table 3).

Youth and HCW emphasized needs for proactive efforts to increase TB awareness, knowledge, and testing among youth, through dedicated outreach campaigns to schools and other community areas frequented by youth. They suggested increasing access to testing at schools through associated clinics serving youth. One HCW highlighted the need for increased youth access to TB preventive therapy. Several participants noted that social media campaigns could be effective for increasing TB awareness among youth. Youth described ways in which increasing TB knowledge could help reduce stigma. Importantly, HCWs noted that opportunities are easily identified to expand services to reach youth where they are in their communities and to provide testing and prevention services that meet their needs, yet that critical funding and investments are required.

For youth who have been diagnosed with TB, areas for intervention as part of person-centered care included providing in-depth health information and supporting close follow-up and adherence to treatment. Participants also strongly emphasized youth vulnerabilities and needs for economic and nutritional support during TB treatment. Some also described needs for mental health counseling.

Participants expressed that current TB services are not well-designed for youth and need tailoring to better meet youth needs. Identified opportunities for improvement spanned multiple areas of service delivery. These included incorporation of some TB services within comprehensive youth clinics. Some favored youth-dedicated TB clinics or spaces, though current space constraints and limited resources were recognized. Several participants indicated that youth-dedicated times within TB clinics would be feasible. Some recommended that the TB clinic environment should be made more welcoming. Participants recommended that HCWs should be friendly, warm, and encouraging, and that they should have training in providing adolescent care.

Youth and HCWs felt that youth TB survivors would be best placed to promote TB awareness, testing, and treatment adherence, including through peer support and community-based interventions. Youth TB survivors were considered best able to have open and clear communication with other youth, supporting health education and optimal adherence. Participants considered ways that youth peer support could be incorporated both in the clinic and over mobile apps or social media. Further considerations include implementation of peer support groups, and of youth “fun days”—social gatherings at which youth could meet other youth who have had TB—for both health education and social support.

Participants emphasized needs for routine post-TB follow-up which includes health education and assessments, including for PTLD and TB reinfection. Some recommended a differentiated care approach, under which those with PTLD symptoms may have regular clinic visits. Many noted that travel reimbursement is needed for follow-up visits. Several described youth needs for psychosocial support to overcome persistent stigma, isolation, and losses that they experienced from TB or TB treatment. They recommended that support be delivered through peer groups, potentially in community settings. Some spoke to the need for a “mentorship” model to support youth to overcome physical, social, and educational setbacks they experienced from TB.

## Discussion

This qualitative study comprehensively investigated youth experiences, care needs, and areas for intervention across the TB diagnosis, treatment, and post-TB care cascade, from the perspectives of youth and HCWs at two major referral hospitals in East Africa. Youth experiences in TB care—particularly delayed diagnosis, stigmatization, adherence barriers, and economic and food insecurity—have important implications for TB transmission, treatment outcomes, and post-TB health. Multiple opportunities exist to improve youth TB outcomes and support post-TB health and wellbeing. Youth and HCWs readily identified solutions that they considered acceptable and feasible in this setting. Through these interviews they detailed the landscape of gaps in care, opportunities, and solutions for youth with TB. From their perspectives, implementing care models and interventions that meet youth needs will require dedicated programming, meaningful youth involvement, implementation research, and sustained investment.

Participants described several youth vulnerabilities that are not adequately addressed by current TB programming and policy.^1^ These included heightened risk of delayed diagnosis, limited awareness of TB and personal TB risk, and limited access to testing under generally passive case-finding systems, sometimes resulting in advanced illness. There is a need for increased youth-focused active case-finding efforts, such as school-based or mobile outreach to increase TB awareness and testing and reduce stigma. Previous studies in Kenya have found that active case-finding strategies among youth yield high TB prevalence.^8,37^ Given the importance of school-based transmission, dedicated approaches to contact-tracing and case-finding are particularly urgent in these settings.^38,39^

TB case-finding efforts should incorporate attention to common pathways to youth TB diagnosis. Ill youth frequently present to school nurses, pharmacies, or lower-level care facilities. Efforts are needed to empower individuals in these facilities/cadres with information about TB and TB testing. Public health messaging should be posted in these spaces to increase awareness that a persistent cough could be due to TB and that TB testing is free of charge. Participants in our study described major delays in accessing TB diagnostics. Recent WHO-recommended TB diagnostic strategies facilitating community-based diagnosis—including near-point-of-care molecular testing, tongue-swab sampling, and sputum pooling—may be particularly impactful in youth congregate settings, including schools, and at lower levels of care.^40^

Several challenges emerged in youth TB treatment, including adherence barriers, TB/HIV stigma, isolation, poor mental health, food insecurity, and financial barriers to transportation or healthcare. Youth were recognized as especially vulnerable to these social, economic, and psychological challenges. Youth may not be developmentally ready to fully adhere to treatment, yet they are often tasked with managing it independently. Youth need strong social support, yet TB stigma and infection precautions leave them isolated and disrupt relationships. Further, youth are economically vulnerable—reliant on caregivers for financial support or living a “hustling” lifestyle—and many did not have enough food during TB treatment. HCWs in TB clinics strove to provide support, but faced inadequate resources. This study adds to others describing major needs for investments and programming to implement youth-friendly TB services.^25,28,41^ Participants highlighted the feasibility and potential impact of youth peer support, dedicated clinic hours, and HCW training in adolescent care. Comprehensive person-centered care must also include nutritional and financial support, as well as mental health care and referral.^42^

Youth who completed TB treatment described overall relief from its physical, social, and mental burdens. Several, however, expressed ongoing challenges from stigma, isolation, livelihood impacts, and ongoing physical limitations or symptoms for which they lacked access to care. Recent studies have lung impairment in 57–65% of adolescent TB survivors,^16–19^ demonstrating the high prevalence of PTLD affecting this age group. As participants in this study discussed, emerging care models will need to address youth needs for accessible, friendly, and appropriate care.^43^ Innovative service delivery approaches might include peer-, mHealth-supported, and/or differentiated care models. Research and programmatic efforts in post-TB service delivery should include direct engagement with youth in implementing youth-responsive care.^35,44,45^

Lending further support to this, youth and HCWs highlighted the crucial role for youth themselves in identifying care gaps and solutions. Meaningful engagement of youth is needed throughout the planning and delivery of youth-focused services.^44^ Interventions leveraging youth-led supports across the TB and post-TB care cascade are highly favored by youth and HCWs. Building youth-responsive models of care, with youth input and feedback across planning and delivery, can help ensure that youth needs are addressed effectively to improve outcomes in this group.^46^

There are several limitations to this study. The relatively limited number of interviews, while appropriate for this qualitative analysis, limits the scope of the findings. Only one youth participant with TB had HIV co-infection, limiting exploration of experiences related to TB-HIV. We conducted the study at TB clinics in two major public referral hospitals in East Africa, so experiences among patients at these facilities may differ from those at different facility types. Conversely, the study was strengthened by triangulation of interviews across youth and HCWs, as well as by the inclusion of sites in two different countries in East Africa.

## Conclusions

Youth experiences during and after TB treatment are marked by limited care access, prevalent stigma, burdens of TB illness and its treatment, and mental health challenges, as well as needs for treatment support and social support. Youth and HCWs described multiple opportunities to address youth barriers to care and unmet needs. They spoke unequivocally about their preferences and needs for interventions leveraging youth involvement and youth-led supports. There is a clear need for youth-specific programming, policy, and research, and for dedicated investments to address this vulnerable group. Designing and implementing youth-focused interventions and programming are essential next steps for improving TB care and outcomes for youth.

## Figures and Tables

**Figure 1:**
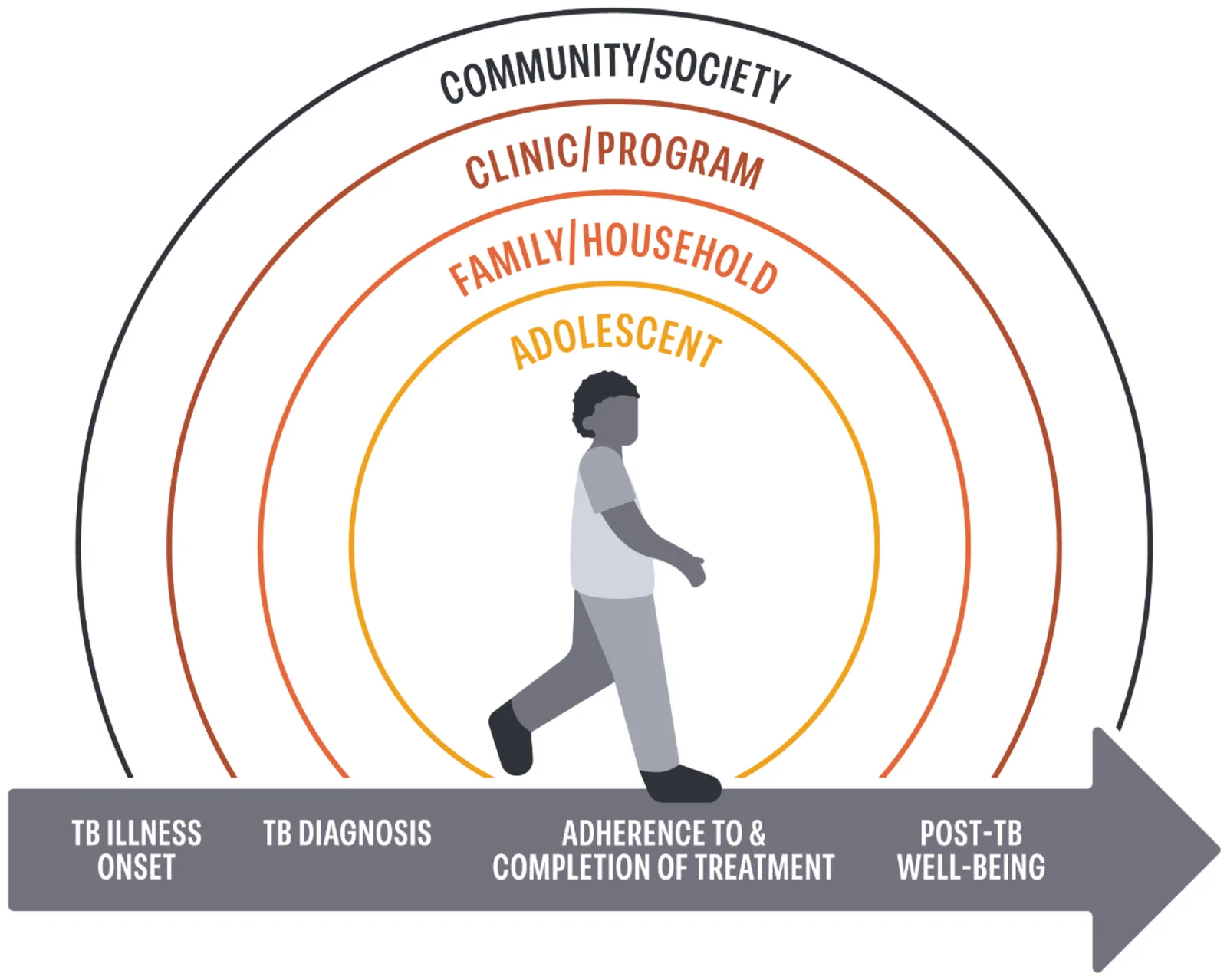
Conceptual framework for investigating youth experiences and care needs across the TB care journey.

**Table 1:** Sociodemographic characteristics at TB treatment initiation among interviewed youth with pulmonary TB, n = 14.

Characteristics	N (%)
Age at TB treatment initiation^a^, years (median, IQR)	22.0 (20.0–23.0)
Sex	
Male	8 (57)
Female	6 (43)
Living with HIV	1 (7.1)
Months from TB symptom onset to TB treatment initiation^a^, weeks (median, IQR)	3.0 (1.6–4.8)
Months from TB treatment initiation^a^ to interview date (median, IQR)	6.6 (6.0–7.0)
Successful TB treatment outcome for current course	14 (100)
Experience of TB recurrence^b^	2 (14)
Study site	
Eldoret	9 (64)
Mbarara	5 (36)
Area of residence	
Rural	7 (50)
Peri-urban	5 (36)
City/urban	2 (14)
Occupation	
Student	5 (36)
Employee, private sector	2 (15)
Cook	1 (7)
Storekeeper	1 (7)
Other	3 (21)
Unemployed	2 (14)
Educational level	
Primary	3 (21)
Secondary	8 (57)
Post-secondary, university, or post-graduate	3 (21)
Monthly income	
< 40 USD	4 (29)
≥ 40 and < 80 USD	2 (14)
≥ 80 and < 200 USD	6 (43)
≥ 200 and < 400 USD	1 (7)
Do not know	1 (7)
Main source of income	
Dependent on someone else’s income	9 (64)
Self	5 (36)
Vital status of biological parents	
Mother living	12 (86)
Father living	10 (71)
In a relationship	5 (36)
Has biological children	3 (21)
Residence	
With immediate family	5 (36)
With extended family	5 (36)
At school	2 (14)
Living independently	2 (14)
Primary caregiver	
Self	4 (29)
Mother	3 (21)
Father	2 (14)
Aunt or uncle	3 (21)
Grandparent	2 (14)
Living with primary caregiver	7 (50)
Roles of primary caregiver in TB care	
Providing transportation fare for the youth to attend clinic	9 (64)
Bringing the youth to clinic visits	4 (29)
Supervising the youth taking medications	3 (21)
Picking up prescribed medications for the youth	3 (21)
Bringing the youth to the hospital when sick	3 (21)
None / not involved	1 (7)
School attendance status	
Attending school	4 (29)
Not in school due to lacking funds	6 (43)
Has completed school	4 (29)

Abbreviations: HIV, human immunodeficiency virus; IQR, interquartile range; TB, tuberculosis; USD, United States dollars.

a. Refers to index course of treatment (i.e., the course initiated on enrollment in the study).

b. One participant had previous episode of TB prior to the current course. One participant experienced two episodes of TB recurrence after their index course of TB treatment (i.e., the course initiated on enrollment in the study).

**Table 2:** Youth experiences and care needs across the TB care journey, with selected excerpts.

Themes	Excerpts
Cross-cutting themes across the TB care journey (from TB diagnosis, treatment, and post-TB health)	
Limited access to care	*“But then whenever it was cold that is when you could find that I’m really coughing but when I am in the sun it gets better so, from want I thought I always thought that it was pneumonia, so I went and was given pneumonia treatment it took sometimes and was a bit better, but still I started coughing again, so I came back, so it got to some point when I could cough them I spit blood, so I felt like it is now getting complicated. So I made a phone call at home and told them that it was like this and this, I was told to go to the hospital, so I went back and then the pharmacist told me [again] that it is the pneumonia that reoccurred.”* – Young woman 1
Specific challenges at school	*“[Youth] of those ages are in school, and if you happen to see even one of them and then by bad or good luck, they test positive for TB. First of all, there is that stigma and so they do not want their fellows to know. And not even the teachers want the information to be known. By the way, that is such a major challenge, when a child tests positive the teachers do not want it to be known, and even at home the parents do not want the information to be known at school. And maybe they have been going to school and the TB has spread there already, you see in that case even if screening is to take part in such a place probably many other people can be helped, but then they do not agree… Maybe when someone is in so much pain that is when they will come.”* – CHP1
Prevalent stigma	*“Even myself I was scared of my neighbors, because the moment they will know [about the TB diagnosis] everyone would close their door, so you will just be like neglected, people are looking at it like as if it is something like an abomination to them, as in they just look at you like as though you are cursed, they don’t want to associate with you anymore. That is where you get yourself like… you just feel like these people don’t want you anymore.”* – Young woman 1
Burdens of TB illness and its treatment	*“Every tablet I would swallow, I would throw up, lost my peace, had no appetite to eat or drink. I hated myself because I was on medication, yet my health was not getting any better. I reached a level of wishing to die.”* – Young woman 2
Mental health challenges	*“And then number two the issue of adherence is that one [when] there is depression… Like around one year since I came here, [there was an adolescent that wanted] to go abroad but then was found to have TB… Normally you will be told to first take your medication. Looking at it, it is again a long time. So, we normally offer them counseling… That is why normally leads to asking themselves ‘why.’ You know adolescents like hanging out in numbers, so they ask themselves, ‘why me, why was I the only one who got infected and not my other friends?’”* – Pharmacist 1
Needs for treatment support and psychosocial support	*“If you look at the dynamics of young people, the adolescents and young adults, the dynamics are purely different because some of them are still under the care of other people. But there are those maybe who have started taking on the roles of adulthood as early as possible and still, it doesnt’ take away the fact that they are also affected by other social dynamics. So, the diagnosis of TB in the youth, or adolescents, it involves a lot of players, including the schools where they are studying from, the health care system where they are coming, and also their households. The diagnosis of TB in this age group is totally hard, I would say, because sometimes adolescents may come and they may [reject a TB diagnosis] because they know that TB kills… On the side of health care workers,… you need to gain their trust, so they believe in what you are telling them, and so that you can help them.”* – Medical officer 1
**Themes by stage in TB care journey**	
**TB diagnosis**	
Delayed TB diagnosis	*“It took about 5 months [to be diagnosed with TB] while taking tablets for cough, not knowing what was in my chest. I would get them from [the] health center. … I was in pain; I was getting worse day by day. When I inquired about X-ray, I was told I could only find it in [town]. That is how I came for further medical tests, but I did not know that I had acquired TB. I had only come for check-up and to find out why the chest was paining me so much. When I arrived, I was tested, and it was discovered that TB and it’s in advanced stages.”* – Young woman 2 *“There is a knowledge gap of signs and symptoms of TB to know that this one has TB among the patients and even the health workers because when those school nurses get such patients, they think it is normal cough. So, getting a patient in that age and is a student, sometimes it is always late, when they have started coughing blood and when the lungs have really been affected too much. And others in that age, if they are not at school, they are working, and they think that work is better than seeking for treatment and so that age group, the patients are really diagnosed late and to keep them on treatment is also not easy.”* – Registered nurse 1
Delays in diagnosis while at boarding school	*“The cough started when I went to school. … I just went to the chemist and asked for a flu tablet which I was given, yes, I took it but then there was no change. So, when I saw no change, I decided to just forget about it because I could not come saying that I bought this and that, I took some time just assuming it, you are there, you go to class, you do whatever… When I came home, I still had that persistent cough. Then my mom said that this cough of mine was not normal, it has been long, maybe it’s time we go see a doctor. We went to the doctor [the next day] and that is when I was sent here. The reason why my mom was suggesting that is because I had a friend and they also had that cough. Later it was said to be TB, so I didn’t know that maybe I could have been affected or …I don’t know how you get affected by it. This friend of mine also got treatment and they got well. A year had gone by, so you see you cannot imagine that it could be the same thing. So I came here, got treated and I was told that I had TB, they just talked to me, and I was told that it has treatment.”* – Young woman 1
Caregiver support for diagnosis	*“In terms of diagnosis for this age group it is probably empowering parents, the guardians, the caretakers who might be the ones who are able to notice some of these things even before [a youth] can say you know this cough is too much I need to go to the hospital. Thinking about it they are likely to seek care, health services through their guardians, so I think this might be the best advocate for them.”* – Medical officer 2
Competing needs and priorities	*“You know we as youths, we normally find it a challenge to come, you see now we tend to feel like we are wasting time but it’s not time wasting. You are going* *to check how our body is doing.”* – Young man 1 *“If they are students, they have stigma, they will fear to take TB drugs while at school, when they are just working, they will forget, they will leave their drugs at home and go to work. Others, they dont’ have time to go to health units, they come very late and very sick. That is the problem at that age.”* – Registered nurse 1
Limited TB knowledge and awareness in the community	*“For us as the youths you might find that there are those who are scared and there are those who are not scared. There [are those] whom you can tell… that they are supposed to go to some specific place for testing, but you will realize that they are scared, they are thinking that it is something like HIV and something of the sort. So the most advisable thing is that the doctors can explain to them. Like if they can get some time and go to some area and then explain to the people there. You see, not everyone knows about TB. There are those who know and there are those who do not know. You see, like for my case now I know it, when you have TB, you are supposed to go to the hospital for testing. The first thing is that you will be tested for HIV, then after you go for the other testing and then you are offered TB medication.”* – Young man 1
Disbelief regarding possibility of TB	*“I went to a school clinic and in fact the doctor tells me that I come for [a TB] test but I didn’t consider it was it and I just ignored it. Then some several days go… I wasn’t even thinking it was it. I was even saying that I have never come across people with that disease so it can’t be me. So I decided to just keep it and I went to [my internship]. I went and worked there in fact after that the body gained energy and there is not even other symptoms. But back those days I experienced headache, chest, in fact chest pain and even the other day I fainted. In fact I went for a shop and buy some drugs… after buying them trying to walk to the place that I live,… I fainted.”* – Young man 2
TB as an opportunistic infection in youth with HIV	*“Most of the opportunistic infections that affect young adults living with HIV, one of them is TB, which is really affecting them so much because of poor adherence. And in a percentage, let’s say, out of 10, maybe [two or three] are having TB. So it’s a bit of challenge to them, because they dont’ really understand where and when to get TB. They dont’ know the symptoms. They tend to [take a break from antiretroviral treatment], then later on, when they come back to medication, that is when after assessment by the clinician, that is when they are diagnosed with TB.”* – Peer mentor 1
Stigma contributing to delayed diagnosis / Need for information and support	*“With this age set you find that there is that, how do I call it, I mean to hide, they don’t want to come out openly, so diagnosis is difficult. They prefer to go over the counter and purchase there drugs which will not help them. So, you find it takes time before it is actually diagnosed that is why they come when it is full blown.”* – Registered nurse 2 *“Some fear the hospital thinking that when it is discovered that they are suffering from a certain kind of sickness that can affect them. What you can do is to always have consistent conversations, counseling and encourage them to check their status and give them assurance that even if it were discovered that they are sick, there is still hope for them.”* – Young man 3
**TB treatment**	
Needs for health information, counseling	*“First, [youth] should be educated on signs and symptoms of TB and drug adherence because some may even forget to take the drugs.”* – Young man 4 *“They need a lot of information. Yeah, once they get such a diagnosis. Their scope of knowledge in that condition is still a bit low in a good number of them, so ideally, they need a lot of communication from the word go. They need some counseling. To know much about management and how to take medication, their adherence.”* – Clinical officer 1
Treatment burdens	*“They fear coming to the hospital, the pill burden, the drugs are too much and too big.”* – Other HCW 1
Adherence challenges	*“The challenge for adherence, is that they have stigma, they dont’ want to be seen that they are taking drugs. Another challenge is that they have no independence and if they are at school, they have problems of getting real treatment. When they are at work, they fear to lose their job, and they also lack sensitization in their communities where they are coming from. You see, really sensitization is not too much so that the patients know that when I have ABCD, it might be TB, so, they come late for diagnosis, and this [also] affects adherence. When they improve, they think they have cured and end up pausing treatment and fail to finish their dose.”* – Registered nurse 1 *“You know when you take this medicine you are relieved of the pain so there is one who is tempted and tells himself, ‘I am ok, I am healed,’ because he feels fine… You know if he stops… now from when he stopped to when he restarts there will be a gap, he will take but it won’t work so after 6 months he will realize there was nothing he was doing. He will restart and then start taking them and then [become lost to follow-up] like before so he never completes his [course]. You know… if someone experiences critical [illness from TB] he will never mess up with the dosage. If he witnesses someone that does not go home because of TB… he knows if he plays around with that medicine, he loses his chance, so he says why can’t I use this chance well so that I don’t get there. Just talk to them… If someone is talked to, he will know oh it is so bad, it is not the way I think. And he will learn from there.”* – Young man 5 *“These young people I think what they only require is privacy… so that they are able to adhere to medication. And also support… If their family is not able to support them in health care… we work as the health care team, we are supposed to give them support.”* – Registered nurse 2
Mobility as a barrier to adherence	*“It’s hard for some of them to complete the treatment. They move a lot, so they relocate from one place to another and remember that is also the age group where they are in colleges they are in school so if not handled well most of them don’t even finish treatment… That is where poor adherence is witnessed in that particular age group and I can only say it’s because of the activeness that they have moving from one place to another place, sometimes forgetting to carry their drugs and sometimes just giving up like they have taken these drugs for long and they now feel they are well.”* – TB coordinator 1
Challenges managing TB at school	*“We find someone who is going to school, going to classes, sometimes some will just miss [treatment], with the excuses that they were doing an exam, or it was an exam week so they did not come, or they will be coming so much late which is almost missing them.”* – Registered nurse 5 *“For me what motivated me was a guy in our school, he missed for like a week he became very sick to an extent that he couldn’t walk, he was just vomiting so I had to inquire from the nurse what the problem was. He told me that he had skipped the TB drugs for a week and that is the reason as to why I couldn’t miss taking my drugs… At that time the drugs were being taken to school and he had missed, the drugs had not been delivered… That is a challenge that affected me also while I was at school… For me, one thing that should be done, someone should be the one picking the drugs and if the school has a nurse then the nurse has to be informed and should be the one managing that so that it becomes easy for tracing if someone misses the drugs at some point.”* – Young man 4
Challenges getting enough food at school	*“Sometimes school just has its own complications, just challenges like you know when you were taking medication, you were supposed to be balancing food and what not, but then you know at school the comrade life is normally challenging. So, you just had to sort out yourself and see how best you can do it.”* – Young woman 1 *“The challenge is that when they are at school, they do not feed well, they feed only on black tea, posho and beans. These drugs also have side effects, they make one feel dizzy and weak, so this makes it difficult for them to take but when they are at home, it is much easier.”* – Registered nurse 3
Challenges with TB stigma at school	*“My biggest challenge was because I was at school and when they would realize that I had TB, they would treat me differently so like you were to find a place where you can take the drugs so that people cannot judge you. I used to delay behind for some time when people were going for supper so as to take the drugs.”* – Young man 4 *“Very many youths are scholars… and especially those that are in boarding schools, they fail to adhere well on their treatment… If this person has not disclosed well to most of his or her friends or to the dormitory leader or the senior mother or to the teachers, it can also affect them to adhere well. Some youth fail to go with the treatment at school or some of treatment is left at home and some is taken at school. So, if he takes for like one month, cough goes away, and so he or she is like, I’m no longer coughing, and then will stop take the treatment. And again, there is this fear of if my fellow colleagues see me taking the drugs, it will be the song of that day, and everyone will start talking. So, discrimination will also be there, they are discriminated like, you are coughing, you are going also to infect us with your cough, and we also get TB.”* – Registered nurse 6
Stigma, avoidance of disclosure of TB illness	*“There is a boy who… was brought here by the father, then he tested positive for TB and was given medication. They did not even tell the mother, when we went to do contact tracing she sounded like she completely did not know about it… The next day in the morning she is the one who called me, “I have found drugs that are looking like this and this and they are hidden.” I told her that with that hiding that means that the drugs are not being taken as required. We talked but then later I called the boy and the father… to them they were finding it embarrassing I told them that there is no shame in sickness.”* – CHP1 *“It is not that free… That [stigma is] still in the society. They start stigma still and they start discrimination we have seen it even in learning institutions that, ‘these students should not come to class because he’s infectious,’ so we’ve been trying to handle it. But we realize it’s still it’s still in the society.”* – Clinical officer 1 *“Another challenge, I was told that this drug you can only take it exactly the time that you take it yesterday (laughs) Now when it reaches this time I have to run from people so that I can go take this drug… So it was a challenge because I did not want people to know… I was trying to be keen that [the time for the dose] does not pass because if it ever passes, I was told that your body will develop that resistance. You remember I have experienced the pain, so if I whatever again and then I experience this pain again. And then I was told that you can transmit this whatever to the other person and I wouldn’t want that. in fact what I have passed through, I don’t want anyone else to go through it.”* – Young man 2
Burdens of TB-HIV co-infection	*“[Youth] do not want their parents to discover that they are suffering from TB. Some of them have HIV but fear to go for treatment and this lowers their immunity. When they get badly off and are brought to the hospital, they approach you secretly and ask that you do not tell their parents about TB and HIV but to tell them that they just have chest pain. We even have patients on ward who do not disclose what they are suffering from, they keep telling them that they just have chest pain. All we do is counsel these youth and encourage them that the only way they can take treatment as prescribed is to disclose to their parents or choose any other member in the family that they trust who we can have a conversation with.”* – Other HCW 2 *“Majority of the ones that I have encountered, they [had discontinued ART] for almost a year or six months… So when they get really, really sick, that is when they come to the clinic. For example, there’s a client who [stopped ART] for almost a year or so, and then he came in so weak, so down, weight was totally lost. That was when the first thing the, I think he the clinician did, was to first see how he has been acting, how he has been eating, that is when they said… he was diagnosed with TB… It’s almost a year or so for him and [now] there is another one who just decided to leave [ART] for two months, and just like that she was infected with TB.”* – Peer mentor 1
Stigma and mental health burdens	*“Mentally, you know as a sick person, when someone tells you something it hits you and makes you wonder… so, there is that mental torture, when you have TB, there is that mental torture, like you wonder… you think about what someone has said and the state you are in, and you feel like you had rather even die… rather than stay in this world, because you feel like you are a burden to other people, you see?”* – Young man 6 *“Sometimes you just sit, maybe by yourself, and you keep asking yourself, ‘how did this happen,’ just so many other things, and then you know it had also this thing like… like say people have known about it, so you see sometimes the way people talk you feel like it hurts, so you keep asking yourself questions like, ‘did I… did it request to be sick and whatnot,’ just such like things, so you realize hat that thing does hurt you.”* – Young woman 1
Financial burdens during treatment	*“During the time that I was admitted, for us at our home, family, we only depend on my dad, so that is why I decided that I look for something to do so that I can at least help him because … he is the only one helping everybody. … So that time* *when I was sick, we did not manage to pay the whole [hospital] bill, though at work they helped me a bit with ten thousand shilling then we paid half of it and there is still a pending bill. That is the problem that I have.”* – Young woman 4
Providers seen as not friendly	*“You find at some point when you go to some clinics you get harassed because you’ve not gone with anyone… The way he was talking to me… This guy was telling that he gave me drugs the other day and I have come for more. I showed him the card that was showing that I had taken all the drugs, and I was wondering whether there was a problem if I finished the drugs or maybe should we pay [implication – a bribe] or what.”* – Young man 4
Needs for privacy and confidentiality	*“Mostly, the youth need privacy, when they come for treatment, they always don’t want to join the line so they shouldn’t be noticed by their fellow youth and so, they always want the take first priority and to be taken to the private room for counselling and be given treatment. Doing this, makes them come back next time and even swallow their medicines with ease.”* – Other HCW 1
Need for adherence support	*“If he’s a youth and you will explain to him and give them like an educational video about TB… What can also help them is to give them treatment supporters, like someone to help them, be near them and keep on reminding them about taking their drugs. And they need more of education, and this education is about the disease and about the need of completing treatment.”* – Registered nurse 1
Need for adherence support from school nurse	*“Most of the youth in that age category are in school and adherence may not be easy. As I mentioned that disclosure is very important, we can go ahead and attach either the teacher that will help or the school nurse if the school has so they can continue giving this youth treatment on DOT.”* – Registered nurse 4
Family-level challenges impacting TB care	*“We diagnosed a 16 year-old girl, after we had given her drugs, the parents reached home and took the child to a witch doctor thinking that the girl was bewitched yet she had serious TB… I kept doing follow up on this girl but they were not picking. Then later they picked up and told me that the girl was badly off to which I advised they bring her back. By the time she was back, she had serious TB complications. I restarted her on treatment, tried to counsel the parents and they accepted she starts on treatment again, when they took her back home, she eventually died…. We have to keep doing health education everywhere. I felt bad for that beautiful girl.”* – Registered nurse 7 *“Those [youth for whom] I’m having issues are those patients who are, for example, orphans, maybe they cannot afford or their parents died… because of HIV… Orphans have challenges when it comes to adherence… They tell you, maybe they relocated. They are outside our catchment area. Okay. You cannot reach them. Okay, so you cant’ confirm with them. You cant’ communicate even with them. Maybe they dont’ even have a phone. Maybe no one takes care of them, actually. I know I’ve got to.”* – Registered nurse 8
Competing needs, economic barriers / need for caregiver support	*“I would say that adherence is good for those who are still under the care of someone, or parents, at least those ones, their adherence is good. But these ones who are in the active labor force, who start to work early, we have higher incidences of lost to follow-up… Most of these people who are still under close watch by their relative, the guardian, it is easy trying to improve, and their adherence is fair compared to these ones who are staying alone. They are starting life or they are working somewhere, it’s a little bit challenging.”* – Medical officer 1
Importance of Engaging caregivers / treatment supporters	*“For me if someone is not schooling then they should find somebody close. Such people so that they disclose to them for them to remind them to take drugs* *and monitor them.”* – Young man 4 *“The challenge with these TB drugs is their side effects. When you are talking to patients, they keep complaining that we have never taken these drugs and we do not know their magnitude. The medicine makes them weak. Most of them have nothing to drink nor eat. If only they could open up to their family members, they would be helped… If the family members know what they are going through and they show them love, they easily recover because they are always mindful. You find that when one has prepared porridge, they will leave some for the TB patient to use to take their drugs.”* – Registered nurse 3
Role of encouragement from HCWs	*“If it is discovered that [a youth has TB], they need to first be counseled. This* *enables them continue with treatment—no matter what their fellow youth say,* *they can never be discouraged or diverted.”* – Registered nurse 3 *“What I learned [from the COVID-19 pandemic], young people cannot depend on themselves in taking… medication. So these continuous check-ins need to be really improved. Like for the healthcare side, we really have to keep on calling… It’s really good to be a brother’s keeper, like, oh, how is my client going on?… It really helps. If we neglect them, if we leave them like, you are now an adult, you know what time to take, they tend to forget, because human beings are [prone] to error. If people are not following me up then it is not so serious, I can just stop and I dont’ know the risk of even what happens when I stop, yeah. [Providing] more knowledge to them should really improve.”* – Peer mentor 1 *“You just advise them about taking medication until when they will have finished. Because you see most of us when we feel like we have gotten a bit of relief we normally tend to feel like we are already well. Even for my case, if I wasn’t advised by the doctor to finish my medication, I wouldn’t have been on treatment right now, I could have felt like I have gotten a relief and so there is no point really.”* – Young woman 3 *“The first is counselling, the doctor I got gave me counselling, he said that those medicines were good, and that I would not die because my TB had not gone beyond, and that I was lucky not to have HIV, that I was going to be okay. So, when I took those words in my heart, I gained confidence, and I started taking drugs, then I could recall those words whenever taking drugs.”* – Young man 8
**Post-TB health and wellbeing**	
Physical health: recovery	*“When I was told that I was done with TB treatment, I was very excited and I started doing my usual activities, I had been limited from playing football by the doctor. When I was told that I was done with treatment, I resumed playing football and I did not feel anything painful in my respiratory system. I also used to isolate myself so that I do not spread TB to others, but I am now free. I have also gained some weight, the other time bones were almost covering my face but when I look in the mirror, I see change every day. I also told my mother who told me to buy enough chicken and eat it and celebrate.”* – Young man 7
Physical health: continued impairments	*“You become breathless like that daily since, if I carry something heavy, or if I sit in a cold place, I feel the stabbing in my chest.”* – Young woman 4 *“After completing treatment, whenever I get flu or cough, they take long to recover, and I don’t know why. Even sometimes I get weak. … Sometimes I get scared and think that I may have acquired TB again because I am not recovering from flu and cough… I have had challenges because I got this problem two months ago but because am at work they can’t easily let me leave to come this side. By the time I get the permission it really takes time and even when I come this side and get the treatment, they write for me drugs that am supposed to buy yet I don’t have money.”* – Young woman 5 *“[I had] some pain in the chest, although I was told that it won’t go away at once but it will take time… I would say economically I am [challenged] because I havent’ gotten the confidence of doing the hard jobs because of the chest pain.”* – Young man 4
Mental health and social wellbeing: recovery	*“I no longer [have suicidal thoughts] because what was bothering me is no more. I have never had that thought again.”* – Young woman 2 *“When you complete your medication, you return to normal. You again associate with people you stay with, you go back to your job. Everything returns to normal, you love yourself. Even when you are nearing completion you see some changes like gaining some energy, loving yourself and you feel normal like any other person.”* – Young woman 5
Mental health and social wellbeing: continued challenges	*“Even those who don’t feel stigmatized, maybe other people might discriminate them, even after going through, finishing the drugs for the infections period, so when they go and continue taking their treatment when they are less infectious, people might also still think that they are still infectious, so it is also traumatizing.”* – Registered nurse 9
Care needs: Post-TB follow-up and physical health assessment	*“Even someone calling you and they are like, ‘is everything okay?’… yeah, you know it’s so nice, somebody feels like I finished my medication, but these people are still there for me… They should be checking on them after the medication… Because we are not many, people who suffer from TB… in as much as its airborne (laughs) we are not that a large number so they can just be checking up on them.”* – Young woman 6 *“In the first place we dont’ have post-TB care. So that’s not there… Once initiated, it is a very good idea… We need to follow-up… post-TB patients, across maybe two years, one year… Maybe just clinical aspect just to see how they are progressing. How they are healing and actually to diagnose other conditions that might be associated with post-TB.”* – Program leader *“This [post-TB] follow up should reach other hospitals because from my friend who had TB [was lost to follow-up and] later finished and no [post-TB] follow up was done… Like for my case I still have some problems so they should at least inquire how the patient is doing even post-treatment because respond differently.”* – Young man 4
Care needs: social support	*“You know once you have completed your medication is when now your health is returning, but you need to remember that you have been down in those previous years. Maybe you lost your work, or lost something else, a lot of, maybe let’s say the things you had planned in life, you have lost them. Maybe you have lost friends. So, catching up to get all that back is not easy… For someone who has lost things, they need support… For a youth who has been down all that while, maybe like me for that 1 year, you are just there doing nothing. That is where now there is a challenge… I mean to come back to normal, say just that love [from friends]. You know love is one of the supports, you need that love also, so that you come and fit as a normal human being. So that people see you as normal and now you have fit into a similar league as them.”* – Young man 6
	*“All those services you’ve mentioned are very important because after this person has suffered this TB, they still need to be encouraged that is life is good they can still live well, they can still become what they want to be. Socially you have to re-integrate them back into society especially after a long hospital stay. That they are useful, you have to talk to them. Yeah, it is important.”* – Registered nurse 9
Care needs: financial support	*“On the side of this support maybe financially, you find that when the patients stop medication the support also stops there and these people find some of them are not able to afford for their care for their nutrition part, though they are fully recovered but you might find them relapsing again… So you find me in my opinion I would say that support somehow even… if they get something little to encourage them… Maybe some activities can be introduced to them so that they can be able to fend for themselves in the long run.”* – Registered nurse 2 *“Maybe I can say, support maybe financially things like that it can help a lot… Because that is the main problem for everything… [My] family we only depend on my dad… he is the only one helping everybody. So that time when I was sick, we did not manage to pay the whole [hospital] bill, though at work they helped me a bit with ten thousand shilling then we paid half of it and there is still a pending bill that is the problem that I have.”* – Young woman 4
Care needs mental health support	*“After completion,.. sometimes they get mentally disturbed because these are the ages or the years where they have to get the boyfriends and the girlfriends. When someone recovers and is getting back to the boyfriend and this person is like, no, I got another girlfriend, of course, someone becomes mentally confused… we try to put up counseling and we do the psychosocial counseling, but it is not so much. And sometimes we dont’ even get more information about these clients and because you were treating them and its done, you discharge the patient.”* – Registered nurse 6

Abbreviations: HCW, healthcare worker; TB, tuberculosis.

**Table 3:** Areas for intervention to support youth TB diagnosis, treatment, and post-TB health: themes and selected excerpts.

Themes	Excerpts
Community-level efforts	
Increasing TB knowledge and awareness in community	*“I think the best thing is we explain to the youth about this disease, they should know that this disease you can have it even when boarding those matatus, this is a disease that is transmitted by air… It is a disease that you can get easily… even you cannot know that this person sitting next to you has it… So the best thing I can say is that youths to have that idea about this disease and they should not fear about it. About the disease, it is common, it is common. And like let them not say that it is from me, they should just say that it is from sharing of those common whatever with others… You know youths mostly if there is a place where they gather… In fact, the best testing I can say that you as healthcare [workers] should send some people who can explain them about that… The other place that most youths are, [is at] high school and campus… You just go there and explain to them about this disease and the treatment… and I don’t think there would be any problem.”* – Young man 2 *“Maybe you can reach them through community health workers… so that they give that health talk to the community. But… community health workers, most of the time they give health talk in a baraza (meeting at the area chief’s place). And most of barazas, most of them is adults, parents… So sometimes it doesn’t reach [the youth]. So maybe organize youth programs. That, in the community that can make them come, so that, so that they are being educated about TB, they know about TB, and if they have the symptoms, they come to a hospital. And screening can be done. Youth programs that are free. Specifically… Even health care workers. Organizing programs for youth at the community level.”* – Registered nurse 8
TB stigma reduction in the community	*“The suggestion I have especially for the youth is to go to the community and sensitize them from there because from experience, the youth starting from ages 14 to 28, they have a lot of stigma, they do not want anyone to disclose their status but if you help them understand that they can recover, they can take TB medication. The challenge however is money, poverty.”* – Other HCW 2
Use of social media / apps	*“I think through [social] media… Facebook and Instagram. Most people are there most of the time, especially Facebook, most youths are there, you can post there. So that everyone passing there can see.”* – Young woman 4 *“You know most of the youths are in the social media. In fact, the social media is the best way to explain about this disease… I think posting blogs about the disease so that people can get to know about it. I think the best is to focus more on giving the information through social media because with that it will reach almost everybody.”* – Young man 2
Increasing access to TB testing	*“Possibly having a way of—and this might be broader than just you know for this age group—but diagnosing TB, innovating to find ways of having samples being taken from home and even maybe some element of self-testing so those would be ways of enhancing their care, you know, and breaking barriers.”* – Medical officer 2
Increasing TB prevention	*“We just start prophylaxis to avoid this activity. OK, it will help.”* – Clinical officer 3
TB outreach and screening in schools	*“[The] TB program should actually come up with organized programs, like [each year], you go to a school. It is [on] us now the health care workers to organize. But… you need funds, you need a vehicle to take the health care workers and ferry everything. Like you need someone… a security officer to be there, you need a tent like this… So, it needs support of funds and taking the services… we go to where those people are.”* – Registered nurse 9 *“That age category is a bit of a tricky group, and access would be based on us being able to extend the services to them in schools, and then also in community outreaches… Actually, as a team and as far as contact tracing is concerned, in case we had to come up with an idea on such categories of youth who are working in garages or who are staying in ghettos, for contact tracing instead of telling them to come here at the hospital and yet they are hustling, are we able to extend the services to them during the contact tracing activities. So, it is us to actively go looking out for them [rather] than them coming to the facility.”* – Medical officer 3
**Person-centered care**	
Needs for person-centered TB care	*“I think… we really need a peer support and a psychological support for those who have ever suffered of TB. [They] need to tell their story: ‘If I did it, you can also do it, it’s not a death sentence.’ In addition… you add in psychological support, because being diagnosed with TB is not easy, as people take it. You also need the support, how to survive, how to take what message to take in, what to put away, it really needs to be put into place. Because… here in [facility] is just it’s a normal thing. It’s like going to a bank and removing your money and go back home. You come take medication and then go back, there’s no really good, good support system that you can feel like, honestly, I am cared for here I am needed. The way HIV is treated, also, TB has to be adopted.”* – Peer mentor 1
Health information and close follow-up	*“Keep visiting them and knowing of their welfare, how are they doing, they would even get encouraged to take their medication. You know a young person opens up to the one who is close to them at all times. So, if they notice this certain man or woman has come to visit me, and they are giving me advice and motivating me, then they will keep on taking their medication.”* – Young man 6
Nutritional support	*“[Support so] they can go and buy some food. Yeah, you know at times it is normally challenging for other people when there is no food, and again for their drugs you cannot say that you can just take it and stay on an empty stomach.”* – Young woman 1
Economic support	*“It can be done by… supporting them with transport fee to come and pick the drugs because most of the youth aren’t working and depend on a parent, whereas others have no parents. If we do an outreach, it would be better and good help so that they don’t put in their transport; we take the drug to them, counsel and comfort them.”* – Other HCW 1 *“You know supporting someone, you know that tends to be helpful because to some point maybe someone is financially unstable, so they won’t make it to the hospital to take their medication. So, from that support that you offer people, someone will be encouraged, they will be like… I know it I will be okay.”* – Young woman 1
Stigma reduction	*“You know some, mostly the youths, what they think is that TB is only meant for the older people only, (laughter) so most of the youth… you can meet with someone and they are like, ‘Why me? How?’ and also many people when it comes to TB, they think of HIV and AIDs, so they normally have that shock like, ‘Oh, to mean that I might be having HIV?’ But if they get that awareness and then they get to know how TB spreads, what makes it be like that and again they are explained how they can get over it.”* – Young woman 1
Mental health interventions	*“When it comes to the point of mental health you know at some point there are people who are normally affected, so for them I would suggest that maybe they look for a therapist, like they talk to them, they are to be told that it’s okay, everything will be fine.”* – Young woman 1
**Youth-led and youth-friendly services**	
Youth involvement in the TB program	*“We as young people, like…it’s so easy to get to us, but it’s … how will you get to us. So that involvement. So when you do your things make sure you involve us… when you go it will be easy for [youths] to communicate to [youths], other than the old people… When they see a youth talking to a youth, they can be like, this is serious, it can even happen, we need to listen to this.”* – Young woman 6
Youth roles for outreach and awareness	*“I usually tell people, ‘Now I am an ambassador of TB’ in some way. So, like TB is something that can be treated. Me, I took my medication and now, like, I’m good. So, I do tell people, like… if you do cough, you need to go take that test, it doesn’t cost anything… Telling that person to take the test you are trying to… you are just trying to make this TB look normal.”* – Young woman 6
Youth roles in delivering TB care	*“They could take at least two or three people who have completed their treatment and gotten better… when they explain to them that when you finish your medication you will be okay, that will encourage them, because they will be like, ‘I saw so and so they took the medication, she adhered and now she is okay.’”* – Young woman 1
Youth survivors serving as role models to others	*“I’ll say when, those who come back [to the HIV clinic] from TB, actually, they really have good adherence when they come to the clinic, the best one, meaning they have had a taste of life a little bit… I think that is also the fear that they have, so on this side it supports, it makes you to be more adult and just take responsibility for your health… I would say they… are really, really responsible. They actually are even talking in the support group, they are giving their experience, telling them you dont’ have to try this because it’s not easy. So… they are very responsible in a very good way, sometimes I even doubt and feel like I am not as responsible as them, because they have, they’ve gone through the hard part. So this one cannot really shake them off. We just strengthen whatever we have in place.”* – Peer mentor 1
Needs for youth-friendly TB services	*“I’ll say it’s not youth friendly, there it is just business. There how it runs is you have come to pick your drugs, you come pick them and go, so at least bring in the atmosphere, it’s a warm way. I won’t say that they’re not welcoming, they are, they are really good people working with them and also being as a client or a Clinical Officer. But there is some kind of coldness, I dont’ know. I dont’ know if it’s me alone, but in [the adolescent HIV clinic], I can see youngsters like me. I feel like yeah, I fit in, in this place, but there it’s only me, the cold and just waiting to be called to triage and off I go… If there’s two or three young people there about it, can really boost the others, for them not to feel like they are the only ones suffering from TB.”* – Peer mentor 1
Peer support groups for TB treatment	*“You can form a group within the age bracket now… Support groups, so that when someone is diagnosed with TB you explain to them. So you explain to them you are not the only one taking …you are not the single one taking medication so that there is that encouragement. Because even in the supportive group you can ask where the challenges are, so they will be able to open up.”* – Pharmacist 1
Training providers in youth care delivery	*“Maybe having…training people who can handle youth issues. All the cadres, if it is a physiological counselor, if it is a nurse specialized in youth, if it is a clinician.”* – Registered nurse 9
Youth-dedicated hours and spaces	*“The HIV clinic has done where they have separated these youths from old people and even from the young children, so going forward probably when we have the resources then we can go that way or even still with the same system we can create a day for youth, say like Friday, it is a day for the youth so that they are separated they are treated alone without the old people or the young children. So, but with the availability of resources, yes, we would like to have a youth friendly environment for our youths. Maybe that way their compliance will be boosted.”* – TB coordinator 1
**Post-TB care**	
Health information about post-TB symptoms, avoiding reinfection	*“I think… I don’t think there is a lot of whatever but the support that he or she needs is about maybe being told and being explained to about how to avoid [TB] reinfection. Maybe you can explain this disease and how you can get it. It is not a matter of… that, ‘I have completed these drugs at the moment, I can’t get it again.’ This is a transmittable disease so when you go to this place there is a crowd or where people live or where someone with the disease is you can get it. So, you can even encourage them about this is a disease that can be re-infected so you should be taking care of yourself, so be in open or ventilated rooms or…”* – Young man 2
Routine post-TB health services	*“Like for my case … I still have some problems so they should at least inquire how the patient is doing even post-treatment because people respond differently.”* – Young man 4
Differentiated post-TB care	*“Yeah, so it’s tough, it’s tough convincing someone… who is well to come to the hospital, especially in our setting. But again, I think it just goes back to how well you explain, how well we sort out the social factors that might interfere with patients keeping appointments. One of the ways, again, we go back to telemedicine and sort of like stratifying and, see, someone who is absolutely asymptomatic, this is someone who can be followed up remotely versus someone who is having symptoms, I think you can make a strong case for them to able to be able to come* *to hospital. So it’s more of stratifying even looking at post-TB care. Like, I don’t think all patients are the same. There are some who can have less aggressive follow-up and can be followed-up remotely, while there are those whom it would be important to follow them up in a more, you know, in a closer fashion, in terms of space between visits and also like the contact.”* – Medical officer 2
Educational support	*“I faced challenge at school… Because I was TB positive, they couldnt’ allow me in school, I sat home for eight months without going to school. I went back home while I was in senior two but I didnt’ study, second and third terms, and so, I repeated the class. And in the community, people were saying that I was going to die, that made me hate myself on hearing those words.”* – Young man 8
Mental health support	*“First of all, they might need social support because when they are on TB treatment, they tend to be isolated from home, you find that people do not want to sit near them, when the person touches utensils at home, you find that other family members tend to ignore and distance themselves from that person and that way you find that they are somehow neglected and depressed. Because of that, they might need some psychosocial support because of the negligence and distance their friends are creating between them. This makes them depressed and hence need for support socially. Post-treatment is also important… Every time they come for treatment, they are assessed, we have a section for mental health, so a clinician or a counselor assesses their mental [state] and diagnoses any form of mental illness, be it depression or any other and they are supported. We have that component in our clinic… I think they are benefitting from our services… I think they have been helped because we also have specific counselors who specifically deal with the youth and they tend listen to their issues closely and they are supported. I think they are being helped.”* – Clinical officer 2
Social support	*“You know once you have completed your medication is when now your health is returning, but you need to remember that you have been down in those previous years, so maybe you lost your work, or lost something else, a lot of, maybe let’s say, the things you had planned in life, you have lost them. Maybe you have lost friends. So, catching up to get all that back is not easy… For someone who has lost things, they need support. … You know love is one of the supports, you need that love [from friends], so that you come and fit as a normal human being. So that people see you as normal and now you have fit into a similar league as them.”* – Young man 6 *“Maybe the very group of those who have completed medication, they can be supported… they can do it as a social support group so that they can form that attachment and they might walk together…. Maybe for the young ones, maybe change of place would have been better, not here maybe in a place that is conducive for them they would feel it to be like an outing that would encourage them to come. And it would be also as a group, as a bond then they form the attachment. Then they will walk the journey.”* – Registered nurse 10
Mentorship to youth	*“Well I think,… just like we have said about this age 15 to 24,… these are our future generation, our future leaders, who we should really mentor from an early stage… We should strive to keep them as healthy as possible, both physically and mentally… If we can close them up, I mean bracket them up. Then we shall have done the future generation well.”* – Registered nurse 2

Abbreviations: HCW, healthcare worker; TB, tuberculosis.

## Data Availability

The full interview transcripts generated during the current study are not available for sharing, given the potential for participant identification, the sensitive nature of the research areas, and the terms of informed consent obtained. We note that extensive de-identified excerpts supporting the conclusions of this study are provided with the manuscript.

## List of abbreviations

AMPATH: Academic Model Providing Access to Healthcare
AYAB: Adolescent and Youth Advisory Board
HCW: healthcare worker
HIV: human immunodeficiency virus
IeDEA: International epidemiology Databases to Evaluate AIDS
PTLD: post-tuberculosis lung disease
TB: tuberculosis
TB-SRN: TB Sentinel Research Network
